# Blending Aboriginal and Western healing methods to treat intergenerational trauma with substance use disorder in Aboriginal peoples who live in Northeastern Ontario, Canada

**DOI:** 10.1186/s12954-015-0046-1

**Published:** 2015-05-20

**Authors:** Teresa Naseba Marsh, Diana Coholic, Sheila Cote-Meek, Lisa M Najavits

**Affiliations:** Interdisciplinary Rural and Northern Health, Laurentian University, Ramsey Lake Road, Sudbury, ON P3E 2C6 Canada; School of Social Work, Laurentian University, Ramsey Lake Road, Sudbury, ON P3E 2C6 Canada; Academic & Indigenous Programs, Laurentian University, Ramsey Lake Road, Sudbury, ON P3E 2C6 Canada; Harvard Medical School, Boston University School of Medicine, 25 Shattuck Street, Boston, MA 02115 USA

**Keywords:** PTSD, Substance abuse, Intergenerational trauma, Historical trauma, Two-Eyed seeing, Seeking safety, Traditional healing practices, Residential school, Healing, Aboriginal care

## Abstract

As with many Indigenous groups around the world, Aboriginal communities in Canada face significant challenges with trauma and substance use. The complexity of symptoms that accompany intergenerational trauma and substance use disorders represents major challenges in the treatment of both disorders. There appears to be an underutilization of substance use and mental health services, substantial client dropout rates, and an increase in HIV infections in Aboriginal communities in Canada. The aim of this paper is to explore and evaluate current literature on how traditional Aboriginal healing methods and the Western treatment model “Seeking Safety” could be blended to help Aboriginal peoples heal from intergenerational trauma and substance use disorders. A literature search was conducted using the keywords: intergenerational trauma, historical trauma, Seeking Safety, substance use, Two-Eyed Seeing, Aboriginal spirituality, and Aboriginal traditional healing. Through a literature review of Indigenous knowledge, most Indigenous scholars proposed that the wellness of an Aboriginal community can only be adequately measured from within an Indigenous knowledge framework that is holistic, inclusive, and respectful of the balance between the spiritual, emotional, physical, and social realms of life. Their findings indicate that treatment interventions must honour the historical context and history of Indigenous peoples. Furthermore, there appears to be strong evidence that strengthening cultural identity, community integration, and political empowerment can enhance and improve mental health and substance use disorders in Aboriginal populations. In addition, Seeking Safety was highlighted as a well-studied model with most populations, resulting in healing. The provided recommendations seek to improve the treatment and healing of Aboriginal peoples presenting with intergenerational trauma and addiction. Other recommendations include the input of qualitative and quantitative research as well as studies encouraging Aboriginal peoples to explore treatments that could specifically enhance health in their respective communities.

## Introduction

As with many Indigenous groups around the world, Aboriginal communities in Canada face significant challenges with trauma and substance use [1]. The complexity of symptoms that accompany intergenerational trauma and substance use disorders represents major challenges in the treatment of both disorders. There appears to be an underutilization of substance use and mental health services, substantial client dropout rates, and an increase in HIV infections in Aboriginal communities in Canada [2,3]. This paper provides a review of the literature to explore the feasibility of blending Aboriginal healing practices with the Western treatment model “Seeking Safety” to address issues of self-identified intergenerational trauma with substance use disorders in Aboriginal peoples, utilizing the concept of “Two-Eyed Seeing.” Two-Eyed Seeing [4] is a philosophical, theoretical, and/or methodological approach that recognizes the need for both Western and Indigenous ways of knowing in research, knowledge translation, and programme development. On the other hand, Seeking Safety is the first manualized programme that aimed to treat trauma and substance use integratively [5,6]. Seeking Safety has been used successfully among many minority populations, including African-Americans, Hispanics, and Asian Americans, as well as translated into numerous languages with implementation internationally [1,5-8]. Considering the particular health needs of Aboriginal peoples [3], this paper explores Aboriginal and Western treatments that address both intergenerational trauma and addiction, and the practicality of using the Seeking Safety programme intervention as a part of a Two-Eyed Seeing approach to treatment [9].

Research with Aboriginal peoples is based on relational responsibility, as well as how the researcher relates to the participants. Relational accountability implies that all parts of the research process are related, from the beginning of the research to the end, and that the researcher is responsible not only for nurturing and maintaining this relationship but also for “all your relations” [10]. As an Indigenous woman and not an Aboriginal person to Canada, the first author consulted with a number of Aboriginal researchers and scholars throughout this project—at inception, during research, and while writing this manuscript. She met with several Elders on a weekly basis for 1 year at the Atikameksheng Anishnawbek (Whitefish Lake) reserve in Sudbury with the goal of learning more about Aboriginal communities. She learned that Elders were the carriers of knowledge of both physical and spiritual reality and that they have been educated through the oral tradition. Elders carry credentials that are recognizable in Aboriginal society, especially around ethics and proper community protocols [11]. On the advice given to her by the Elders as well as the third author of this paper, the first author established an Aboriginal advisory group to help guide her through the research process. She continued to meet with the advisory group on a monthly basis. In addition, the first author worked with an Aboriginal supervisor and one Aboriginal committee member who both reside in Sudbury and are members of Aboriginal communities. This literature review was conducted as part of a study that underwent ethical review by the Laurentian University Research Ethics Board. The study received approval from Laurentian’s Ethics Board in May 2013.

While Aboriginal peoples in Canada share the experiences of colonization and the destruction of their cultural practices, it is important to avoid generalizations, as there are three distinct groups of Aboriginal peoples in Canada with unique geographic and linguistic heritages, many subcultures, cultural practices, and spiritual beliefs [12]. There are currently 1.2 million Aboriginal people residing in Canada, of whom 61% are First Nations, 34% are Métis, and 5% are Inuit [13]. In this paper, the term “Aboriginal” refers to First Nations (status and non-status Indians), Métis, and Inuit people as referenced in the Canadian Constitution. The term Indigenous is used interchangeably with Aboriginal, particularly in international contexts. We use the term Aboriginal peoples as a way to respect and acknowledge their shared values, historical residential school experiences, and contemporary struggles with the aftermath of colonization and oppression. In doing so, we also acknowledge the strength and resilience of the Aboriginal peoples in Canada [14-16].

Many Aboriginal peoples suffer from intergenerational trauma caused by more than 400 years of systematic marginalization. According to Gagne [17], intergenerational trauma is the transmission of historical oppression and its negative consequences across generations. Brave Heart [18] explored the concept of intergenerational trauma in her study of the Lakota people. She concluded that most participants in the study displayed many symptoms related to trauma, and agreed with other researchers that trauma experienced by more than one generation becomes institutionalized within the family and community [18]. This type of group trauma, both cumulative and psychological, can have a profound impact on health and has been proven to affect not only the lifespan of an individual but the lifespans of generations that follow [19].

According to Duran [20], Aboriginal peoples underutilize available mental health and addiction services. Also, there are significant treatment dropout rates for those who do seek care and support. There has been concern that Western treatments and conventional psychology have failed to address the needs of Aboriginal peoples because they do not understand traditional spiritual and healing methods that continue to persist in many Aboriginal communities [21-24].

In response to this underutilization of health services, health-care professionals have moved toward more holistic, culturally sensitive approaches and have endeavoured to blend Western health-care practices with traditional Aboriginal healing practices [22-25]. The blending of Aboriginal and Western research methods, knowledge translation, and programme development has been called Two-Eyed Seeing [9]. Two-Eyed Seeing refers to learning to see from one eye with the strengths of Indigenous knowledge and ways of knowing, and from the other eye with the strengths of Western knowledge and ways of knowing. Two-Eyed Seeing then encourages the use of both these eyes together, for the benefit of all [26]. For many practitioners, care incorporates Sweat ceremonies, a cultural practice performed in a heated, dome-shaped lodge that uses heat and steam to cleanse toxins from the mind, body, and spirit; smudging, the burning of sacred herbs in a small bowl to purify people and places; drumming, the use of ceremonial drums and songs as a way to connect with the Creator and spirit; Sharing circles, a healing method in which all participants, including the Elders, are viewed as equal and information, spirituality, and emotionality are shared; traditional healers, who use a wide range of activities, from physical cures using herbal medicines and other remedies to the promotion of psychological and spiritual healing using ceremony; and Elder teachings [23,27,28]. This holistic view of mental health and addiction not only ensures that care is culturally relevant but also encourages connection to the community [23,29].

In a recent study that reviewed the literature on the use of interventions to treat substance use disorders in Indigenous populations, Rowan et al. [30] found 19 studies in the United States (58%) and Canada (42%) that integrated Western and culturally based services in both residential and outpatient programmes. The authors reported that the results showed benefits in all areas of wellness, as well as the reduction in substance use in 74% of the studies [30]. In another study, Jiwa et al. [31] reviewed 19 opinion, review, and programme description articles from six quantitative, three qualitative, and two mixed-methods studies. They identified that the literature on community-based substance use programmes emphasizes the importance of viewing substance use disorders through a sociocultural, spiritual lens. In other words, the incorporation of sociocultural, traditional practices and community-based models proved more successful. The authors also agreed that there is a paucity of evaluation, research, and outcome data for these programmes.

## Review

### Historical overview of intergenerational trauma

The term historical trauma, also referred to as cumulative trauma [18], soul wound [20], and intergenerational trauma, originated from research into the experiences of Holocaust survivors and their families [32-35]. It refers to the cumulative emotional and psychological harm experienced throughout an individual’s lifespan and through subsequent generations.

There is an important distinction to be made between intergenerational trauma and post-traumatic stress disorder, which is a psychological disorder also caused by exposure to trauma. Brave Heart [18] stated that although post-traumatic stress disorder (PTSD) is adequate to describe the depth and effects of cumulative trauma, it is too narrow in scope and therefore fails to adequately address complex Aboriginal experiences. She further stated that the theories of historical intergenerational trauma and historical trauma response accurately described and can help others to understand and acknowledge massive cumulative trauma.

It is well documented that the intergenerational trauma experienced by Aboriginal peoples is linked to experiences at residential schools [14,17,36,37]. The Government of Canada implemented Indian Residential Schools from 1831 to 1996 [38]. These schools were operated by Christian churches and were encouraged and financed by the Canadian federal government [24]. Aboriginal parents believed that their children would receive education, but in truth, the Canadian government implemented the schools to solve “the Indian problem” [39,40]. These schools encouraged and forced students to repress their Aboriginal culture and practices. Many scholars have researched the impacts of residential schooling on Aboriginal peoples and shed light on why so many Aboriginal peoples suffer from trauma, violence, self-harming behaviour, and addictions [41,42]. The brutal experiences in these schools were reported by survivors as a force that shaped their lives and future parenting styles. Internalized oppression and neo-colonialism became the hallmark of so many as they expressed hatred toward themselves, their culture, and traditional values and beliefs [37,43] leading many to later struggle with identity issues [33-35]. Chansonneuve [44] explained that some residential school survivors express their grief as lateral violence directed toward family and community members, thereby creating intergenerational cycles of abuse, which can resemble many of the experiences at the residential schools [45].

Duran [20], an Apache/Pueblo Native American psychologist, concluded that the Aboriginal patient suffered a “soul wound” through multiple losses sustained from colonization, particularly from forced attendance at English-speaking boarding schools. Duran [20] and Brave Heart [46] connected the idea of a soul wound with historical trauma and observed success in using culturally based workshops and interventions when treating Aboriginal clients: “Intervention strategies that have been useful in dealing with the soul wound have been effective in many ways. People have engaged in the healing process and have made use of traditional forms of healing.” They agreed that drawing on Indigenous knowledge and worldviews offered therapists, healers, and health-care practitioners a valuable way to assist clients to work through their traumatic experiences. Similarly, many Aboriginal Elders referred to the symptoms of trauma as spiritual injuries, soul sickness, soul wounding, or ancestral hurt, and encouraged clients through their teachings to use traditional medicines and healing to heal the soul [20].

Intergenerational trauma is the most common term used to describe the systematic trauma suffered by Aboriginal peoples [47-49]. Wesley-Esquimaux and Smolewski [50] introduced the Historical Trauma Transmission model, describing it as a system in which “trauma memories are passed to next generations through different channels, including biological (in hereditary predispositions to PTSD), cultural (through storytelling, culturally sanctioned behaviours), social (through inadequate parenting, lateral violence, acting out of abuse), and psychological (through memory processes) channels”.

### Challenges of treating substance use disorder in Aboriginal peoples

There is no doubt that addictive behaviours and substance abuse have taken a terrible toll on Aboriginal populations in Canada, contributing to far greater incidences of accidents, disease and illness, violence, and death compared to the rates typically found in the general population [14]. Corrado and Cohen [51] completed a review of case files of former Aboriginal residential school pupils who had undergone clinical assessments in British Columbia. Of 127 case files reviewed, 82% reported that their substance use disorder behaviours began after attending residential schools. In addition, 78.8% of the former Aboriginal residential school pupils had substance use disorder, almost three times the rate of the general public [19]. This coincides with research that shows a link between post-traumatic stress disorder and alcoholism [52,53], as well as a link between discrimination and the likelihood of American-Indian adults meeting criteria for substance use disorder [54]. Corrado and Cohen [51] noted that “alcohol abuse is strongly associated with historical loss” (p. 413), and Haskell and Randall [48] agreed that “this is a very significant finding because it delineates a connection between the use of alcohol as a form of coping or numbing feelings by people attempting to deal with overwhelming current and/or historical traumas” (p. 71). This research concurs with the findings of Whitbeck et al. [55] which stated that the prevalence of substance use disorder, behavioural problems, and depression were approximately two times greater for Aboriginal children aged 10 to 12 years old [54].

While it is clear that intergenerational trauma has affected the mental health of Aboriginal peoples, it is important to identify that the impact varies from community to community [22,56]. For example, Chandler and Lalonde [56] note that, while certain Indigenous or First Nations groups do in fact suffer dramatically elevated suicide rates, such rates vary widely across British Columbia’s nearly 200 Aboriginal groups: some communities showed rates 800 times the national average, while in others suicide is essentially unknown. Finally, they demonstrated that these variable incidence rates were strongly associated with the degree to which British Columbia’s 196 bands are engaged in community practices that are employed as markers of a collective effort to rehabilitate the cultural continuity of these groups.

There is an agreement among several researchers that culturally sensitive assessment tools and interventions are needed to enhance healing from substance use disorders in Aboriginal peoples [14,54,57]. Importantly, there is also a need to consider treating substance use disorders and the effects of trauma concurrently since they are so closely connected [14,54].

### Treatments that address intergenerational trauma with substance use disorders

#### Aboriginal spirituality and healing practices

The key to healing following the experience of residential school abuse and its intergenerational effects lies in the area of reclaiming identity [58,59]. Many authors have argued that reclaiming Aboriginal identity means recovering traditional values, beliefs, philosophies, ideologies, and approaches and adapting them to the needs of today [17,29,57,60]. This reclamation of traditional culture can encompass both individual and collective identities and can be sought by way of traditional health methods. According to the World Health Organization [61], traditional medicine is:

The sum total of knowledge, skills, and practices based on the theories, beliefs, and experiences indigenous to different cultures, whether explicable or not, used in the maintenance of health as well as in the prevention, diagnosis, improvement of treatment of physical and mental illness.

The *Report of the Royal Commission on Aboriginal Peoples* defined traditional healing as:

Practices designed to promote mental, physical, and spiritual well-being that are based on beliefs which go back to the time before the spread of Western ‘scientific’ bio-medicine. When Aboriginal Peoples in Canada talk about traditional healing, they include a wide range of activities, from physical cures using herbal medicines and other remedies, to the promotion of psychological and spiritual well-being using ceremony, counseling and the accumulated wisdom of Elders [22,25,62] (p. 348).

Thus, there appears to be a consensus among researchers and practitioners that restoring traditional healing practices and knowledge is a pathway to both empowerment and health for Aboriginal peoples and communities. Aboriginal spirituality, premised on the principles of trust, sharing, respect, honour, and acceptance, cannot be divorced from traditional healing methods. This spirituality has sustained Aboriginal peoples throughout their existence, including the period prior to the introduction of residential schools [62,63]. However, in order to achieve this goal, the traditional knowledge once practised in historical Aboriginal societies must be revived as a treatment philosophy when treating substance use disorders and trauma in Aboriginal peoples [31,64].

Duran and Duran [57] suggested that culturally based approaches to the treatment of trauma and addiction should have many facets and must include multidimensional approaches with an emphasis on both intervention and prevention strategies, which are essential for improving the mental health status of Aboriginal peoples. Duran and Duran [57] also emphasized the need to restore balance in all areas of life for Aboriginal peoples by providing bilingual education and encouraging Aboriginal traditions, customs, and spiritual teachings as a means of increasing self-esteem.

Attention to culture and/or traditional healing has been reported as a successful element in substance abuse programmes designed for Aboriginal peoples [54,65,66]. For example, a quantitative study of a self-identity enhancing approach to preventing substance abuse stressed the importance of enhancing self-concept through cultural affiliation [67]. Through surveys and self-reported data, these authors found that engaging off-reserve Aboriginal youth aged 14–19 years in cultural activities yielded lower rates of substance use disorders compared with the control group that had not participated in cultural activities [67]. Similarly, the Round Lake Treatment Centre in British Columbia implemented a programme that emphasized cultural awareness through healing circles and family involvement. Evaluation of results from this programme from 1991 to 1995 indicated that most participants were no longer struggling with substance use 2 years after completing the programme [67]. Also, between 1999 and 2003, about half the adult population (200 people) from the Montagnais-Innu village of Nutashkuan in Labrador participated in nature camps located on their ancestral hunting territory. This programme, which was strongly influenced by traditional Aboriginal spirituality and led by Montagnais-Innu healers and non-Innu psychologists, resulted in a subjective assertion by these leaders of a “steep drop in the rate of consultations for domestic violence” [66] which is often exacerbated by substance use disorders.

#### Blending Aboriginal and Western healing methods through Two-Eyed Seeing

One way of incorporating Aboriginal traditional healing practices into treatment for substance use and intergenerational trauma is through the concept of Two-Eyed Seeing. Two-Eyed Seeing recognizes Indigenous knowledge as a distinct and whole knowledge system that can exist side by side with mainstream (Western) science [9]. Two-Eyed Seeing asks of us, in a respectful and passionate way, to bring together our different ways of knowing and to use our understanding and wisdom to bring about healing [9].

The application of the concept of Two-Eyed Seeing advocates for inclusion, trust, respect, collaboration, understanding, and acceptance of the strengths that reside in both Western and Aboriginal worldviews [68]. Through collaboration and the demonstration of mutual respect in worldviews, Two-Eyed Seeing encourages Aboriginal peoples and health-care providers to develop a relationship of mutual cultural respect, wherein the benefits of both worldviews are acknowledged as beneficial in the healing processes. Also, the incorporation of traditional healing and cultural involvement can further develop a sense of identity in individuals suffering from intergenerational trauma and substance abuse, which is extremely important for recovery.

Two-Eyed Seeing emerged from the teachings of the late spiritual leader and healer Chief Charles Labrador of Acadia First Nation in Nova Scotia. He stated, “Go into the forest, you see the birch, maple, pine. Look underground and all those trees are holding hands. We as people must do the same” [9]. Chief Charles Labrador’s concept of Two-Eyed Seeing was first discussed in the literature in 2004 by Elder Albert Marshall from the Eskasoni Mik’maw Nation in Nova Scotia [69]. Elder Albert spent most of his childhood and teenage years in the Indian Residential School in Shubenacadie, Nova Scotia. He was profoundly affected by this experience, and it led him on a life-long quest to connect with and understand both cultures as an integral part of his own healing from intergenerational trauma [69]. Marshall felt that students in the Integrative Science co-learning journey at Cape Breton University would benefit from meaningful collaboration and communication with Elders and teachers in Aboriginal communities [9].

According to Stewart, blending Aboriginal and Western treatment methods involves the incorporation of Aboriginal traditional healing practices and traditional healers; the presence of Elders in treatment programmes; the involvement of local communities, drumming, smudging, and Sweat ceremonies; and the participation of non-Aboriginal treatment providers in community events and ceremonies [27,65,70]. This blended model of care has the potential to increase the rate at which Aboriginal peoples access mental health services and decrease programme dropout rates. Furthermore, a blended approach could strengthen relationships between Aboriginal and non-Aboriginal service providers and could encourage cultural understanding [23,27,28].

Blending these two approaches can be challenging, yet a worthwhile task when done in an ethical and culturally safe way. Western approaches to knowledge are characterized by the individualized ownership of knowledge and efforts to quantify for the purposes of generalizability. Aboriginal approaches to knowledge are contextualized, relational, and owned by the community [71]. In the Aboriginal worldview, knowledge and the knowers or learners are intimately connected, meaning that they are connected to everything and everyone around them, casually referred to as “all our relations, be it air, water, rocks, trees, animals, insects, humans, and so forth” [10]. In the Western sciences, this is usually not the case. Because of this connection, Aboriginal knowledge is more accurately described as a way of living in nature [72] that is strongly place-based; the goal of Aboriginal knowledge is to become open to the natural world in body and spirit [71].

Limitations for blending Aboriginal and Western treatments could include the risk of continuing to oppress Aboriginal peoples and knowledge. Historically, Aboriginal communities were forced by academics or government agents to participate in research with little or no understanding of the purpose or practice that would be undertaken. The outcomes of these research projects were often disrespectful, misguided, and harmful [11]. Thus, the way in which approaches are blended and facilitated must take into account the values, practices, and beliefs of Aboriginal peoples in a way that is respectful and inclusive. In addition, many researchers and treatment providers made statistical generalizations by treating Aboriginal peoples as if they were one large group without recognizing their diversities [15,31]. In order to avoid this risk, clinicians and researchers must recognize that each group of Indigenous peoples have cultural concepts that are specific to that particular group [58,73-75].

Other potential limitations of blending approaches include the reality that many Aboriginal communities lack the resources to recover and revitalize their language and culture. Policy should acknowledge traditional knowledge as a critical component to success of preventative and intervention strategies for Aboriginal communities, yet currently this is not the case [58,59]. In addition, another barrier to the implementation of blended research and interventions is the awareness of rural and northern issues across provincial government organizations, including the social determinants of health; Aboriginal health; existing policies, programmes, and services; and the shortage of Aboriginal doctors, researchers, and other health-care professionals in the north. There may be substantial issues implementing Two-Eyed Seeing Indigenous decolonizing methodology without these vital resources and systems of support [65,76,77].

#### The Seeking Safety counselling programme

The Seeking Safety programme aims to increase the coping skills of participants with the goal of reducing the chance of relapse by emphasizing values such as respect, care, integration, and healing of self [78]. Participants work to reduce suicidal and self-harming thoughts and behaviours, including the urge and desire to use substances and other unsafe behaviours. They also work to remove themselves from unhealthy relationships in order to gain a sense of control and healing [5]. Group participants develop skills such as grounding, an act of mentally (and sometimes physically) linking oneself with the earth or another source of power like the moon, stars, another element, or any other natural source of energy to calm the mind and thoughts; joining the present making sure you are present in the group or circle; and changing what can be changed to reduce the severity of their urge to self-harm [6]. Seeking Safety is considered a first-stage therapy, and as such, the primary goals of treatment are abstinence from substances and acquiring coping skills to obtain personal safety [78].

Hien and colleagues [79] compared the effectiveness of Seeking Safety and relapse prevention with non-standardized community care treatment for 107 urban, low-income, treatment-seeking women. Participants’ substance use and PTSD symptoms improved during the Seeking Safety and relapse prevention programme, but not in the community care treatment. Najavits and Hein [8], in their recent review of the literature on treatment studies for co-morbid substance use disorder and PTSD, showed positive outcomes on multiple domains. They found that the Seeking Safety programme was the only treatment outperforming a control on both PTSD and substance use disorder. Seeking Safety is also listed as having strong research support by various professional entities based on their criteria sets, including Level A by the International Society for Traumatic Stress Studies, and “strong research support” by Divisions 12 and 50 of the American Psychological Association [6].

What makes the Seeking Safety model unique is that, unlike traditional Western treatment programmes that emphasize more of a medical model or exclude the aspects of treatment, Seeking Safety encourages spiritual discussions by offering a philosophical quote at the beginning of the treatment’s group sessions [8,78]. Seeking Safety also bridges this gap by including discussions about safety, cultural continuity, gentle language, and teachings about the genesis of intergenerational trauma and addiction [5,8]. Furthermore, the Seeking Safety treatment model encourages the treatment of trauma and addiction in an integrative way. Rather than treating these conditions separately, Seeking Safety emphasizes a holistic and integrative approach that addresses trauma and addiction simultaneously. Seeking Safety incorporates the inclusion of the mind, body, spirit, and self-awareness during treatment, as well as connection to community through emphasis of the utilization of community resources. Thus, the perspective of Seeking Safety is convergent with Aboriginal traditional methods because traditional methods include the values and concepts of holism, relational connection, spirituality, cultural presence, honesty, respect, and connection to land and all of creation [27].

#### Similarities between Seeking Safety and an Aboriginal traditional healing approach

The Seeking Safety manual is written in a form that is culturally sensitive, empowering, and with an understanding language that addresses the needs of the clients [5]. The Seeking Safety model is positive, strengths-based, respectful, supportive, and collaborative [6]. These elements constitute what may have been missing from other mainstream treatment models that led to large attrition rates with Aboriginal participants [25,27,55].

Furthermore, the manual pays detailed attention to safety and self-care, not just for the client but for the clinician as well. These two factors are often closely monitored within Aboriginal communities, particularly when traditional healing practices are utilized. For instance, during a Sweat lodge ceremony, the Elders may explain and teach about the entire process before anyone enters the lodge, advocating that the teachings are for the safety of everyone [44,80-82]. Another example of the collaborative nature of Aboriginal healing is that the Elders never enter the lodge alone [44,82]. There are always helpers present to aid the Elders and to make sure that the participants are kept safe during the ceremony [46]. In sharing her experiences with the Elders and Aboriginal communities, the first author was encouraged to write this manuscript to explore whether it would be possible to utilize the model in Aboriginal communities [46].

**Figure 1:**
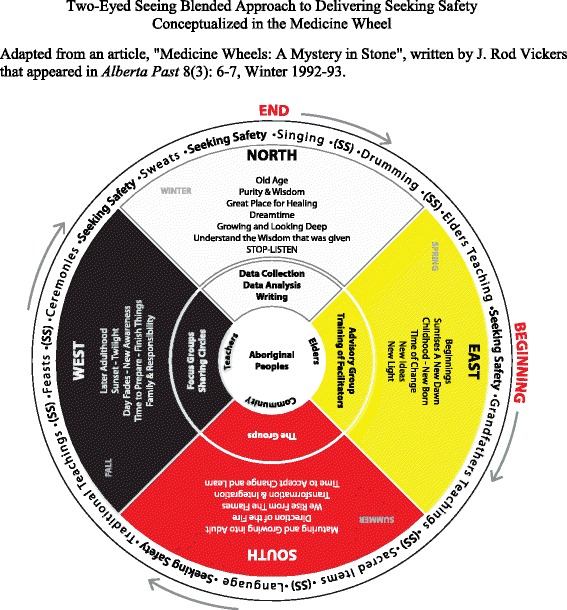
Two-Eyed Seeing blended approach to delivering Seeking Safety conceptualized in the medicine wheel. Adapted from an article, “Medicine Wheels: A Mystery in Stone,” written by J. Rod Vickers [86].

In exploring how Aboriginal traditional healing practices could be blended with the Seeking Safety model in the treatment of intergenerational trauma and substance use disorders, a visual conceptual model of the blended approach was developed (see Figure 1 which is further explained in the Appendix). The Seeking Safety programme can be offered in a group format in 1- or 2-h group sessions [5]. Sharing circles can replace these groups, because Sharing circles are a method that is familiar and comforting for some Aboriginal peoples in Canada, who have knowledge of this practice [80]. Healing circles and learning circles are also sometimes used to describe Sharing circles. They are used as part of ceremony as a way of healing [81] and are increasingly used by Indigenous researchers [80].

Also, the presence of an Elder in the Sharing circles would be an important healing practice to add to Seeking Safety, as Aboriginal peoples have long recognized the role of the Elder as integral in the healing process. Elders’ skills, knowledge, and ability to help individuals restore balance in their lives have earned them significant roles within Aboriginal communities [36]. The Elder’s role in the Sharing circle is to focus on the positive identity of each and every one in the circle and to help develop the connection to the spiritual world through their teachings.

The Seeking Safety manualized programme also provides information about topics through handouts that aim to teach participants a variety of skills. The majority of topics address the cognitive, behavioural, interpersonal, and case management needs of persons with substance abuse and PTSD [5]. To adhere to cultural sensitivity, the material could be conveyed verbally. Aboriginal facilitators are encouraged to use language that respects their cultural values and beliefs. For example, a session on anger can be explained through the role of the sacred fire. Some Aboriginal peoples believe that we are all our own fire keepers and must make sure that our fire is taken care of so that it does not destroy us [46]. This would be an appropriate parallel to the Seeking Safety material, which explains anger as constructive or destructive [78].

In the Seeking Safety programme, an inspiring quote is used to start each session [5]. The use of smudging and drumming with singing to open up the Sharing circles can be used in conjunction with the Seeking Safety quote. Smudging is a sacred act that is a part of many rituals. Traditional medicines such as sweetgrass, sage, cedar, and tobacco encompass the four sacred plants. Burning these is a sign of deep spirituality in Aboriginal practices. The cleansing smoke from smudging can be used to purify people and places and calms the central nervous system. A feather or hand-held fan can be used to spread the smoke around, but the hand can be used as well. Drums, on the other hand, represent the heartbeat of the nation and the pulse of the universe. Drums are sacred objects and are often used in healing ceremonies [59,67,71,82]. All songs are seen as honour songs, as their name implies, and are sung to honour the Creator, the ancestors, and particular individuals. Songs can have a profound healing effect [36].

Moreover, making regular Sweat ceremonies available to all participants can be a powerful way to bring forth Aboriginal traditional healing and the Seeking Safety topics, which focus on cognitive, behavioural, or interpersonal aspects of healing. Sweat lodge ceremonies involve the heating of a Sweat lodge to help repair damage done to the spirits of people, their minds, and their bodies. In order to warm the lodge, rocks are heated up in a fire outside the lodge, then brought into the centre of the lodge with a shovel and placed in a pit dug into the ground. Sweat lodge participants sit in a circle at a safe distance from the pit. Sweat ceremonies are led by a properly trained and authorized ceremonial traditional spiritual leader [59,71,82,83]. The Sweat lodge is a place of spiritual refuge for mental and physical healing. It is a place to receive answers and guidance by asking spiritual entities, totem helpers, the Creator, and Mother Earth for needed wisdom and power [31,77,82]. Therefore, the integration of any aspect of the topics can be useful during the Sweat ceremony.

The Seeking Safety programme ends each session with a “checkout” or closing activity. During a checkout, participants provide feedback about their experience during the Sharing circle [5]. They can report what they liked or disliked, what community resources they will use, and what commitment they will make in order to continue their healing. In addition to this, a Grandfather teaching and Aboriginal spiritual and traditional sayings, smudging, and/or prayer could be offered. The teachings of the Seven Grandfathers, also known simply as either the Seven Teachings or Seven Grandfathers, are a set of teachings on human conduct toward others and include the concepts of wisdom, love, honesty, respect, bravery, humility, and truth [83,84]. These teachings could blend well with the Seeking Safety topics on honesty, respecting your time, and commitment. The topics encourage clients to apply these concepts to themselves, their families, and their helpers. For example, clients are encouraged to be honest about their substance use when asked by a child, family member, or Elder. In addition, taking the time to go to the ceremonies and keep appointments with counsellors are all ways of maintaining their commitment and respecting one’s time in recovery.

Furthermore, Elders, facilitators, and/or participants can introduce the utilization of sacred items and sacred bundles. The sacred bundle is considered a very precious possession, which represents a person’s spiritual life and may be placed in the centre of the circle [15,74,83]. A sacred bundle can consist of one or many items. It can be the little tobacco or medicine pouch that someone wears around their neck, or it can be items such as a sacred pipe or rattle that the spirits have given to a person to carry for the people [65,84]. Many Seeking Safety topics can be integrated in the presence of the sacred bundle, for example, the topic about when substances control you and keep you away from your recovery can easily be linked to a traditional teaching about carrying your sacred bundle to help you climb the recovery mountain [36,78,82]. The sacred pipe is also a sacred item that could be used as part of the Seeking Safety programme. Sacred pipes are used during both private and group ceremonies. An offered prayer is believed to be carried to the Creator through the smoke of the pipe. The pipe ceremony and the Seeking Safety topics of having compassion and taking good care of yourself [5] converge, as both practices encourage gentleness and kindness to self. Participants who follow their traditional teachings will be encouraged to bring their sacred items to the Sharing circle to help guide and connect them to their culture and traditions and integrate the Seeking Safety topics [65,85].

Seeking Safety also uses grounding and centering techniques; these are often used together to help traumatized individuals connect with their bodies and elements around them to calm the mind in the group sessions [5]. The facilitator can guide the participants through an exercise encouraging them to focus on different body parts, rooting their feet to the ground or feeling the contours of the chair and connecting to the breath. These could be practised with spirituality and traditional teachings in all of the Sharing circles. For example, a sacred song with drumming and the burning of sweetgrass could be used during the grounding session. The burning of sweetgrass represents kindness and stillness.

Lastly, holding a traditional feast at the onset of the Sharing circles and at the end of the programme would be another traditional practice that is honoured by most Aboriginal peoples. A traditional feast symbolizes and celebrates the gifts from Mother Earth. This is a way of recognizing the spirits and Creator and giving thanks. It also symbolizes renewing the earth by prayers, chants, and dances [73].

## Conclusion

Due to the complexities of symptoms that accompany historical, intergenerational trauma and substance use disorders paired with the chaotic, poor social conditions these clients endure, many clinicians and treatment agencies experience challenges in attracting, retaining, and supporting patients for the treatment [16,31,47,85]. Therefore, research into the treatment of intergenerational trauma and substance use disorders is required so that the challenges that both disorders present can be adequately addressed in treatment modalities. In addition to gaining a better understanding of the issues around the treatment of intergenerational trauma and substance use disorders, there is a continued need to consider treatment and healing programmes that have a focus on Aboriginal peoples and are able to blend Western knowledge and Aboriginal healing practices to better reach and affect those in need. One example of how this can be achieved is through the Seeking Safety treatment programme. The Seeking Safety programme is based on well-respected Western treatment methods, such as an integrative, interpersonal, and educational approach that is very close to Aboriginal healing practices, such as holistic approaches and the use of Elders to bring the teachings about the struggles that people face when dealing with both disorders [31,84].

In terms of future directions, community-based feasibility studies could be implemented to explore the use of Seeking Safety with Aboriginal peoples. Such studies could shed light on this subject and contribute to a better understanding of effective healing modalities in Aboriginal populations. Also, studies where community members are asked how Aboriginal traditional healing practices and Western models could be integrated in their communities could be a valuable contribution to research. The wisdom and teachings from Elders and community members from different geographical areas could be a valuable endeavour in the field of research on this topic.

## Appendix

### Description of Seeking Safety conceptualized in the medicine wheel

This was adapted from an article, “Medicine Wheels: A Mystery in Stone,” written by J. Rod Vickers that appeared in *Alberta Past* 8(3): 6–7, Winter 1992.

The Elders and Aboriginal advisory group, when presented with possibility of utilizing a Western model, Seeking Safety, with Aboriginal traditional healing practices for treating intergenerational trauma and substance use disorders by the first author, advised that the medicine wheel should be used to depict this Two-Eyed Seeing concept. The outer Circle embraces Aboriginal traditional healing practices and Seeking Safety and shows how it can be applied to all people with the guidance of teachers, Elders, and the Seven Grandfather teachings.

The four quadrants in the medicine wheel represent wisdom, the stages of life, Aboriginal teachings, and connectedness [86]. Furthermore, the researcher or clinician can utilize the knowledge of each quadrant as a guide for the Two-Eyed Seeing model. For example, the east quadrant represents spring, new beginnings, childhood, and a time for new ideas. When beginning a research project involving Indigenous peoples, the researcher must be guided by the teachings of Elders, an Aboriginal advisory group, and the community. Indigenous facilitators and community members must all be included in the process from inspiration to expiration.

The south quadrant represents summer, maturing, growing into adulthood, transformation and integration, and accepting knowledge and change. This quadrant could guide the researcher or clinician to begin the preparation for the implementation of the Two-Eyed Seeing and Seeking Safety Sharing circles.

The west quadrant represents spring, adulthood, sunset, a new awareness, a time of preparation, as well as family and responsibility. The west can serve as a guide for the Elders, researcher, the Aboriginal advisory group, and facilitators during the data analysis and drafting the research papers.

Finally, the inner Circle represent the Aboriginal peoples, the community, and the Elders as the most important reason for this integration and blending of Aboriginal and Western knowledge. This would also honour Elder Albert who taught that Two-Eyed Seeing is the gift of multiple perspectives treasured and respected by many Aboriginal peoples. After all, Elder Albert stated that Two-Eyed Seeing refers to learning to see from one eye with the strengths of Indigenous knowledge and ways of knowing and from the other eye with the strengths of Western knowledge and ways of knowing and to use both these eyes together, for the benefit of all.
